# Deficits in the Activation of Human Oculomotor Nuclei in Chronic Traumatic Brain Injury

**DOI:** 10.3389/fneur.2015.00173

**Published:** 2015-08-25

**Authors:** Christopher W. Tyler, Lora T. Likova, Kristyo N. Mineff, Spero C. Nicholas

**Affiliations:** ^1^Smith-Kettlewell Eye Research Institute, San Francisco, CA, USA; ^2^Division of Optometry, City University, London, UK

**Keywords:** oculomotor, dynamics, vergence, binocular, eye movements, fMRI, traumatic brain injury

## Abstract

Binocular eye movements form a finely tuned system that requires accurate coordination of the oculomotor dynamics of the brainstem control nuclei when tracking the fine binocular disparities required for 3D vision. They are particularly susceptible to disruption by brain injury and other neural dysfunctions. Here, we report functional magnetic resonance imaging activation of the brainstem oculomotor control nuclei by binocular saccadic and vergence eye movements, and significant reductions in their response amplitudes in mild or diffuse traumatic brain injury (dTBI). Bilateral signals were recorded from a non-TBI control group (*n* = 11) in the oculomotor control system of the superior colliculi, the oculomotor nuclei, the abducens nuclei, and in the supra-oculomotor area (SOA), which mediate vergence eye movements. Signals from these nuclei were significantly reduced overall in a dTBI group (*n* = 12) and in particular for the SOA for vergence movements, which also showed significant decreases in velocity for both the convergence and divergence directions.

## Introduction

### Subcortical pathways for oculomotor control

The primary form of eye movements as we look around the world is the rapid jumps known as “saccades.” Saccades are well known to be one of the most rapid muscle movements in the body, being completed with a duration of only about 50 ms [from about 20–100 ms, depending on the amplitude of the saccade from 1° to 40°; Ref. (1)]. They have velocities up to about 600°/s in human.

**Figure 1:**
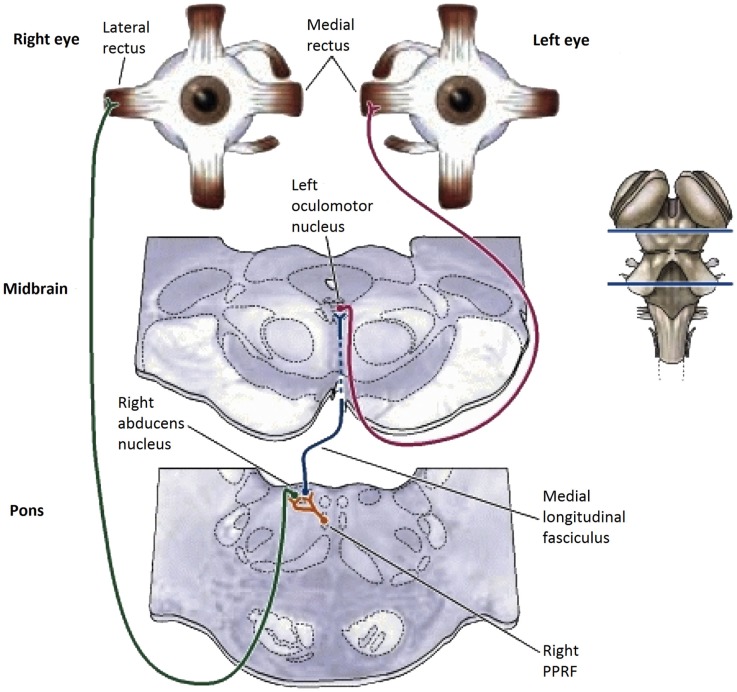
**Summary diagram of the subcortical pathways for saccadic oculomotor control (8)**.

The basic pathways for *conjunctive* eye movements from the cortical motor control regions through the basal ganglia to the brainstem oculomotor nuclei (ON) are well established (Figure 1), both for open-loop saccadic eye movements and closed-loop pursuit eye movements (2–4), although details of the feedback control loops in the basal ganglia are still undergoing progressive refinement (5–7).

In the *brainstem*, there are separate control centers for the vertical and horizontal components of saccades. Each center receives input from both the frontal eye fields and the superior colliculus (which in turn receives frontal eye field input). The horizontal saccadic control centers are the left and right ON in the midbrain, which send signals for an inward eye movement directly to the medial rectus muscles, but control an outward eye movement via the descending pathway of the medial longitudinal fasciculus (MLF) to the paramedian pontine reticular formation (PPRF) and thence to the adjacent abducens nucleus (see Figure 1). The abducens nuclei also contain internuclear inhibitory neurons that project back up the MLF to the contralateral oculomotor nucleus, inhibiting the action of the medial rectus muscles that would oppose an outward eye movement, to allow for the maximum activation that characterizes the saccadic movement. The vertical saccadic control centers are located in the left and right rostral interstitial nuclei of the MLF, with corresponding inputs from the frontal eye fields and superior colliculus.

In human, functional imaging of the brainstem saccadic pathway for blocks of saccades from 8° to 16° amplitude has been reported by Linzenbold et al. (9), using a high-resolution protocol spanning 80 mm in depth at the brainstem level.

#### The Brainstem/Cerebellar Pathway for Vergence Eye-Movement Control

**Figure 2:**
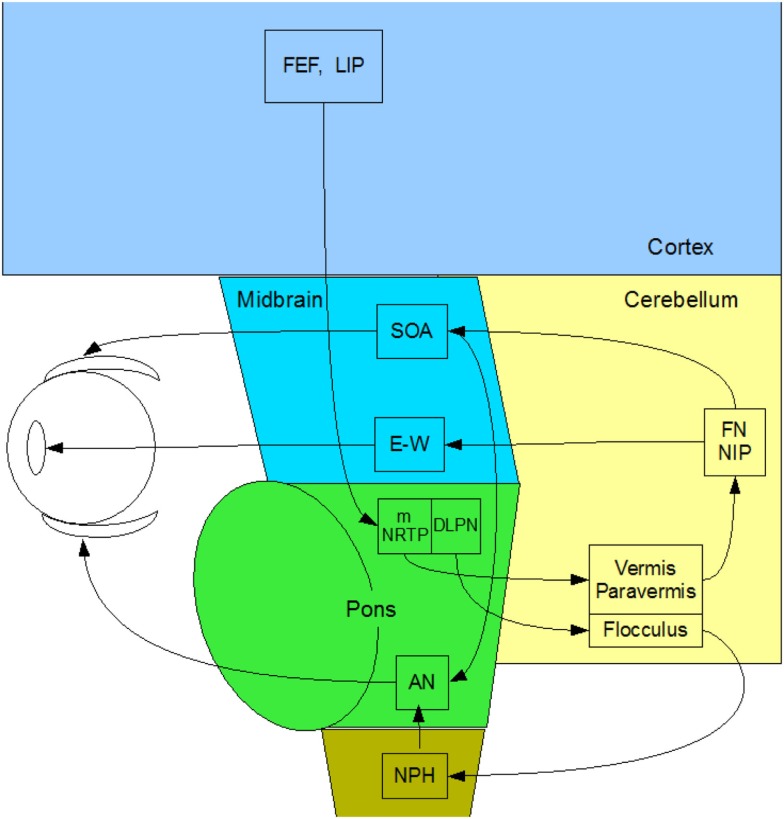
**Summary diagram of the subcortical pathways for vergence control pathways of the medial and lateral recti through the supra-oculomotor area (SOA) and abducens nuclei (AN), respectively, and of the ciliary muscle via the Edinger–Westphal nuclei (E–W), assembled by the present authors from Gamlin (13)**.

Vergence eye movements are the relative (*disjunctive*) movements of the two eyes with respect to each other. They have typical durations from 200 to 500 ms and velocities an order of magnitude slower than saccades of the same amplitude. Remarkably, there have been very few studies of the *human* vergence control system, on which our binocular coordination mechanisms rely. Here, we provide a complete overview of what is known of the subcortical anatomy of the human vergence system, centered on the brainstem and cerebellum (Figure 2). To our knowledge, there *have been no* studies of direct stimulation of the vergence pathways or functional magnetic resonance imaging (fMRI) recording of their relative response characteristics in the human *brainstem*, despite recent work on *cortical* responses to vergence [Ref. (10, 11), with the latter study including one analyzed location in the midbrain], and the discovery of a cortical network responding to dynamic changes in binocular disparity in the human brain (12), which also included direct measures of the cortical activation during vergence movements.

In primates, vergence eye movements are initiated through cortical activation of vergence-specific neurons in the frontal eye fields under stimulation by changes in disparity, accommodation and other dynamic depth cues (in what is termed the “near reflex”). Note that this information is largely derived from studies in the monkey brain [see Ref. (13–18)], partially confirmed by a few lesion studies in the human brain [see Ref. (19, 20)]. These vergence signals project to the pontine vergence control centers (Figure 2), the nucleus reticularis tegmenti pontis (NRTP), and the dorsolateral pontine nucleus (DLPN), which also receives feedback from the cerebellum. The vergence control neurons of the NRTP project to the supra-oculomotor area (SOA) via the cerebellar nuclei and to the abducens nucleus of the pons. The SOA is the immediate premotor area for vergence eye movements, with the neurons of the SOA projecting directly to medial rectus motoneurons (MRMs), and hence, provide predominantly a convergence signal. The projection to the abducens nucleus is less well established, but its output is predominantly a divergence signal in the lateral rectus muscles controlled by the abducens nucleus.

The cerebellar pathway from the pontine NRTP consists of input to the midline vermis and paravermis lobes of the cerebellum, from there down to the fastigial (FN) and interpositus (NIP) nuclei of the cerebellum, and thence, back to the SOA. This recurrent input to the SOA provides a cerebellar feedback signal that helps to coordinate the activations of the various eye muscles and muscles controlling any associated head movements. The fastigial nucleus may be more involved in maintenance of vergence stability and the NIP more involved in divergence movements. The cerebellar pathway for convergence signals is not well established, but may involve signals to the paraflocculus and from the DLPN to another brainstem nucleus – the nucleus prepositus hypoglossi (NPH) of the medulla, which are also involved in the vestibulo-ocular reflex of counter rotation of the eyes against head rotations. The NPH projects up to the abducens nucleus in the brainstem, which projects directly to the lateral rectus motoneurons (LRMs) to control active divergence of the eyes.

Functional magnetic resonance imaging of the brainstem nuclei in human is challenging. A literature search revealed no papers addressing the question of how the function of the ON is affected by mild or diffuse traumatic brain injury (dTBI). The goals of the present study are to measure the normal levels of activation in the brainstem/midbrain oculomotor control nuclei for both saccades and vergence, and furthermore, to determine if there is response reduction in dTBI. Such results will be important both for identifying specific deficits in the mechanisms of oculomotor control, which are critical for everyday tasks such as reading and driving, and for providing the means to quantify such deficits in cases of dTBI, which has wide prevalence in the general population from falls, accidents, and sports injuries, and which is generally associated more with cerebral than with brainstem damage.

## Materials and Methods

### Participants

The human participants consisted of 12 individuals (6 female) with dTBI and 11 age-matched controls (4 female) who met the criteria of corrected letter acuity of 20/50 or better in the best eye with no visible ocular abnormalities. They were recruited into the study on the basis of their participation in a companion behavioral study of eye movements (21). The individuals were assigned to the control group if they had no past history of dTBI events (12 individuals with ages ranging from 22 to 75; mean age: 33.3 ± 13.3). They were assigned to the dTBI group (11 individuals with ages ranging from 21 to 64; mean age: 36.5 ± 14.9 years) if they reported a positive past history of one dTBI events characterized at levels 13–15 on the extended Glasgow Coma Scale [GCS-E; (22)] following the trauma. The participant characteristics are provided in Table 1, where the status for memory deficit on object naming, cognitive status on the clock test, and photophobia by self-report are quantified as 0 for normal, 1 for mild, and 2 for moderate. The time since the dTBI occurrence ranged from 0.2 to 35 years, with a geometric mean of 2.2 years. Nine of the 12 reported either headaches or irritability as a result of the dTBI event. All elements in Table 1 refer to the most recent concussion, except in the fourth column showing the number of concussions previous to the most recent one (Previous Concussions).

**Table 1:** **dTBI participant characteristics**.

Gender	Age	Years since concussion	Previous concussions	LE acuity	RE acuity	Stereo test (arcmin)	Memory deficit	Photophobia	Cognitive status	Symptoms
M	28	6	0	20/40	20/50	0	0	2	1	Headache
F	22	7	0	20/20	20/20	2	0	2	1	Headache
M	22	1	0	20/20	20/20	2	2	2	0	Dazed
M	28	2	0	20/25	20/25	2	2	2	1	Headache
F	75	0.7	0	20/25	20/25	4	2	0	1	Balance
F	40	1	0	20/16	20/16	2	0	2	0	Headache
M	30	8	0	20/20	20/20	2	2	2	1	Balance
M	53	35	0	20/20	20/25	2	2	2	1	Irritability
F	42	0.6	0	20/16	20/20	2	2	0	1	Irritability
F	40	0.2	0	20/20	20/25	2	2	0	1	Irritability
F	44	10	0	20/20	20/20	2	2	2	1	Headache
M	42	1	1	20/32	20/20	2	1	0	0	Irritability

All subjects signed a written informed consent approved by the Smith-Kettlewell Eye Research Institute and the Congressionally Directed Medical Research Program Review Boards in accordance with the Declaration of Helsinki.

### Laboratory procedures

**Figure 3:**
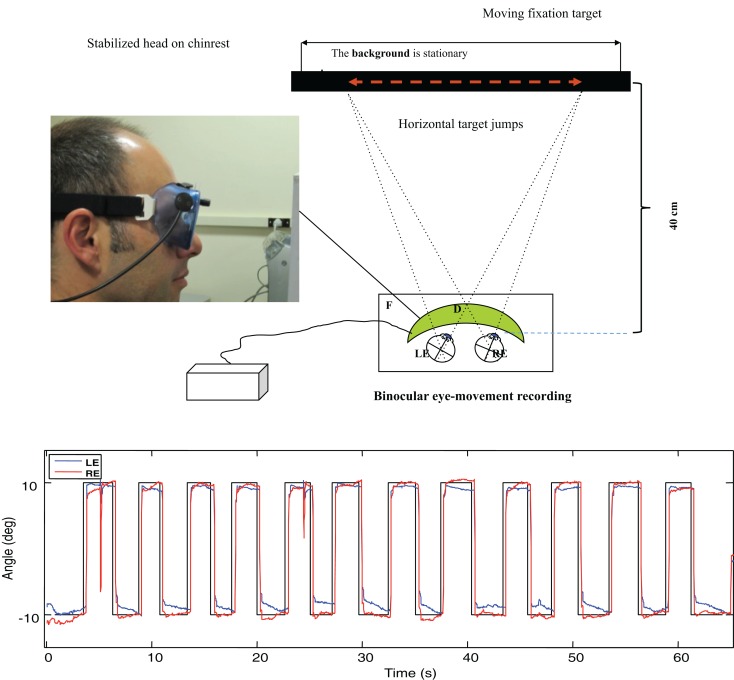
**Upper: laboratory recording set-up for saccadic eye movements**. Binocular eye position is recorded with goggle-mounted infra-red limbal sensors; the target is presented on an LCD monitor. Lower: typical binocular eye-movement recording of saccades with our head-mounted infra-red recording system. Black line – target position for a series of target jumps separated by randomized delays of 2–3 s. Blue line – right eye (RE) trace. Red line – left eye (LE) trace.

Prior to the scans, the participants’ eye movements were measured in the laboratory. The *saccadic* stimulus for the laboratory studies was a 60 s epoch of a 1.25° circle/cross combination jumping between horizontal positions 10° to the left and right of primary (straight-ahead) position on a computer monitor (Figure 3, upper), with the stimulus events repeated with a temporal delay randomized over a flat distribution between 2 and 3 s. The eye movements were recorded with a Visagraph III infra-red limbal eyetracker (Compevo, Sweden). A typical example of an alternating series leftward and rightward saccades recorded in the laboratory is shown in Figure 3 (lower).

The saccades in Figure 3 show a typical rapid time course with a typical latency of about 200 ms with respect to the instantaneous stimulus jumps, with blinks after the first and ninth saccades. Note the fixation instabilities of the order of 0.5° between saccades, and the slow vergence drifts (separation of the red and blue traces) of the order of 1° over 2–3 s in the same intervals, particularly at the left-hand (negative) position. These consistent vergence movements indicate that there is a disjunctive (unequal amplitude) component to the 20° horizontal saccades in this normal subject that needs to be corrected with a vergence to achieve accurate binocular fixation for the beginning of the next trial.

**Figure 4:**
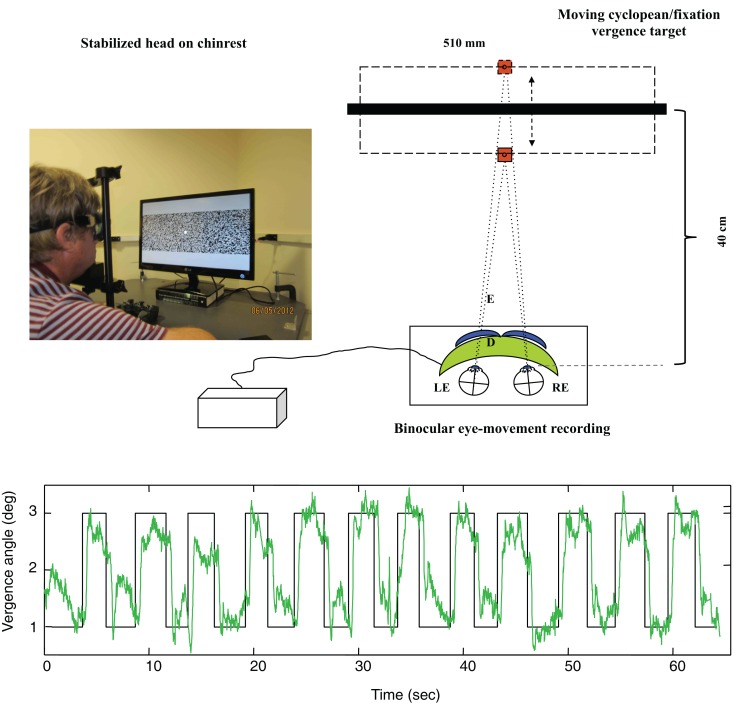
**Upper: laboratory recording set-up for vergence eye movements**. Relative binocular eye position is recorded with goggle-mounted infra-red limbal sensors; the target is a 32° random-dot field presented on an alternate-line polarizing LCD monitor and viewed with polarizers attached in front of the goggles (blue curves). Lower: vergence angle trace for a 1-min series of vergence eye movements alternating far (1° disparity) and near (3° disparity) to a series of disparity jumps separated by randomized delays of 2–3 s. Note that compressed *y*-axis scale compared with Figure 3. There is non-systematic vergence drift of the order of 0.5° between the step vergence movements.

As shown in Figure 4 (upper), *disparity vergence movements* were recorded for a 32° random-dot field with a central fixation target presented on an alternate-line polarizing LCD monitor and viewed with polarizers attached in front of the goggles (blue curves). The stimulus generated disparity vergence stimuli of both the random-dot field and the fixation target alternating between far (1° disparity) and near (3° disparity) in a series of disparity jumps separated by randomized delays of 2–3 s. The example in Figure 4 (lower) shows the vergence angle trace for a 60 s series of target vergence jumps. There are clear vergence movements of about 80% of the target vergence amplitude with a short delay following each target jump. Note that the uncoordinated vergence drift of the order of 0.5° between slightly angled vergence movements.

#### Saccadic and Vergence Analyses

The *saccadic* and *vergence* eye movements were compared between the groups on the basis of five parameters of oculomotor dynamics: onset latency, amplitude, duration, peak velocity, and temporal asymmetry (23). Each parameter estimate is the average of the values for 12 repeats of each eye movement within a 1-min recording session. Outliers beyond 2 SDs of the distribution were removed before calculating the estimates.

#### fMRI Stimuli

The stimuli for the fMRI scans were of two types – *saccadic* targets and *vergence* targets. Due to the small size of the visual display, it was not possible to present activation targets at large angles from fixation. The *saccadic* targets were therefore designated as the edges of the visible aperture of the head coil, which were at a visual angle of about 60° from the central fixation point, and visible only monocularly. The stimulus consisted of a 10° left or right arrow presented at the center of the display every 2 s indicating which aperture edge should be fixated at any given time.

For the *vergence* targets, we needed a real object in space to incorporate all vergence cues (i.e., the disparity, accommodation, and proximal vergence or distance knowledge cues), rather than just a binocular disparity cue. We therefore utilized the blue stripe running along the roof of the scanner bore as the near disparity target, which was at a distance of ~18 cm from the participant’s eyes. For the far cue, we displayed a similar blue stripe on the monitor screen against a black surround, at a distance of 4 m from the eyes. Projecting into the bore, the blue light from this stripe made the physical blue stripe appear low contrast, cueing the participant to look at the high-contrast line on the monitor screen. Conversely, to cue the participant to converge onto the close physical stripe, the display showed a uniform yellow light that illuminated the bore to give the blue stripe a high-contrast appearance. The mirror reflecting the visual display was arranged to have its upper border at the center of the participant’s gaze, so that the visual display was visible just above fixation and the scanner bore just below fixation. The change of vergence thus required a small vertical fixation shift, but the participant was instruction to keep these shifts to a minimum compatible with a reliable vergence shift.

**Figure 5:**
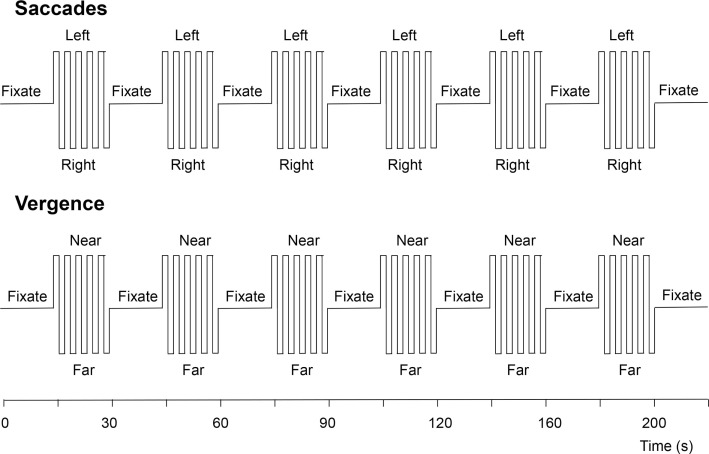
**Functional magnetic resonance imaging protocols for the visually driven saccadic and vergence eye movements**. See text for details.

The vergence change was cued to occur every 2 s, just as with the saccades. The experimental protocol for each eye movement type consisted of six cycles of 20 s of cued eye movements alternating with 20 s of stable fixation, as depicted in Figure 5. The two protocols were run four times each, interleaved between eye-movement types during the session.

### Brainstem imaging

Functional and structural MRI scanning were conducted at the Neuroscience Imaging Center of the University of California San Francisco on a Siemens Trio 3-T scanner equipped with eight-channel EXCITE capability. We took the approach of performing quantitative T2-weighted MRI in the targeted area of the upper brainstem (pons and midbrain regions) in a compact brainstem prescription of 0.7^3^ mm voxels. The MRI used a T2 2D spin echo Fluid Attenuated Inversion Recovery (FLAIR) protocol with TE: 92 ms, TR: 3720 ms, flip angle: 120°, matrix size: 384 × 384, field of view: 180 mm × 180 mm, and slice thickness: 1 mm. The high-resolution functional MRI protocol used a 2D echo-planar imaging (EPI) with 27 slices; TE: 32 ms, TR: 2000 ms, flip angle: 84°, matrix size: 128 × 128, field of view: 200 mm × 200 mm, voxel resolution, 1.56 mm × 1.56 mm, and slice thickness: 1.84 mm. Automated shimming was performed before each EPI session, ensuring better field homogeneity and higher signal-to-noise ratio in the brainstem area.

The functional activations were then processed for slice-time correction, motion correction, and trend removal to filter out the noise from breathing and heart rate artifacts. The activation was specified in terms of the statistical significance (*p* < 0.05) of the signal in each voxel (after Bonferroni correction for the number of voxels in the segmented cortical gray matter). Data were averaged across participants through a 12-parameter affine transform optimization to align the high-resolution brainstem scans, followed by a non-linear warp calculated from the high-resolution 0.8 mm^3^ T1-weighted structural to the MNI152 brain at 2 mm resolution. The 4D functional MRI scans were then transformed into MNI-space for each brain by sequentially applying the same affine transform and the non-linear warp. The average oculomotor activation was largely restricted to the regions of the principal brainstem nuclei, together with patches of visual activation in the anterior calcarine region of the visual cortex, as expected from the strong visual signals generated on the retina from the eye movements.

## Results

### Saccadic Analyses

The laboratory oculomotor dynamics results are of two types: the effects of saccade direction in the non-dTBI group and the differences between normal and dTBI groups. In the non-dTBI group, oculomotor dynamics for the leftward and rightward saccades are in the expected range for saccades of this amplitude. The values for the dTBI group show significantly longer onset latencies, slower peak velocities, and larger asymmetries, but similar amplitudes and durations, as detailed in Table 2. Four comparisons showed small but significant differences at *p* < 0.05 between the control and dTBI groups: onset latency for the right eyes, duration for the left eyes, and peak velocity for each of the eyes. Evidently, the saccadic parameters were susceptible to the residual effects of the TBI event and had not fully recovered their original function.

**Table 2:** **Oculomotor dynamics for horizontal saccades**.

Group	Direction	Onset (ms)	Duration (ms)	Amplitude (°)	Peak Velocity (°/s)	Asymmetry
Control	Left	$\begin{array}{l} \;229\pm 9\\ \begin{array}{l} 234\pm 11\\ 234\pm 7\\ 253\pm 9* \end{array}] \end{array}$	$\begin{array}{l} \begin{array}{l} 62.7\pm 2.0\\ 66.6\pm 1.5\\ 67.4\pm 1.1* \end{array}]\\ \;66.7\pm 1.5 \end{array}$	18.2 ± 0.6	$\begin{array}{l} \;457\pm 32\\ \begin{array}{l} \begin{array}{l} 480\pm 32\\ 469\pm 20* \end{array}]\\ 494\pm 36* \end{array}] \end{array}$	0.05 ± 0.09
	Right			18.7 ± 0.9		0.06 ± 0.11
dTBI	Left			19.6 ± 0.6		0.11 ± 0.12
	Right			20 ± 0.4		0.02 ± 0.10

*Significant difference at *p* < 0.05 on the *t*-test after correction for multiple applications.

### Vergence Analyses

For the laboratory vergence assessments (see Table 3), the parameters were similar for the convergence and divergence directions except for the duration parameter, which was significantly longer for divergence than for convergence in both groups [compare Ref. (23)]. Note that the slow peak velocities for vergence in the Control group were about 1/50th of the values for the saccades. Across the groups, there was a significant reduction in the vergence peak velocity in the dTBI group for both convergence and divergence movements. Other parameters were similar across the groups.

**Table 3:** **Oculomotor dynamics for vergence eye movements**.

Group	Direction	Onset (ms)	Duration (ms)	Amplitude (°)	Peak velocity (°/s)	Asymmetry
Control	Convergence	212 ± 20	$\begin{array}{l} 387\pm 47\\ 458\pm 10* \end{array}]$	1.92 ± 0.24	$\begin{array}{l} \;8.89\pm 0.26\\ \begin{array}{l} \begin{array}{l} 7.86\pm 0.20*\\ 6.81\pm 0.19 \end{array}]\\ 3.06\pm 0.45* \end{array}] \end{array}$	0.21 ± 0.10
	Divergence	251 ± 14		1.87 ± 0.14		0.11 ± 0.05
dTBI	Convergence	208 ± 19	$\begin{array}{l} 343\pm 32\\ 453\pm 35* \end{array}]$	1.63 ± 0.40		0.08 ± 0.08
	Divergence	185 ± 43		2.03 ± 0.36		0.13 ± 0.07

*Significant difference at *p* < 0.05 on the *t*-test after correction for multiple applications.

### Brainstem Oculomotor Responses

**Figure 6:**
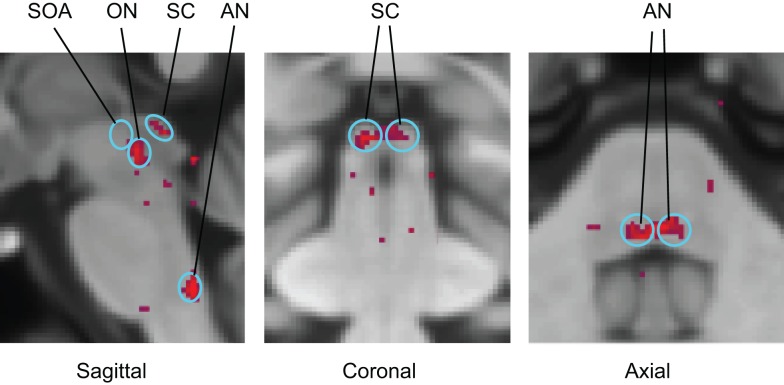
**Identification of the principal oculomotor nuclei to saccadic activation in the normal group: SOA, supra-oculomotor areas; ON, oculomotor nuclei; SC, superior colliculi; AN, abducens nuclei**. All voxel activation exceeding a criterion level of *p* < 0.01 on the *t*-test is shown (red coloration) averaged across the control group after alignment with the MNI brain coordinates, against the background of the MNI152 brain anatomy (gray coloration).

Functional magnetic resonance imaging activation sites in the superior colliculi (SC), ON, and abducens nuclei (AN) are shown in Figure 6. (Activation was also seen outside the brainstem in the lateral geniculate nuclei (LGN), cerebellum, and calcarine cortex.) For vergence movements (not shown), activation was also evident in the SOA.

**Figure 7:**
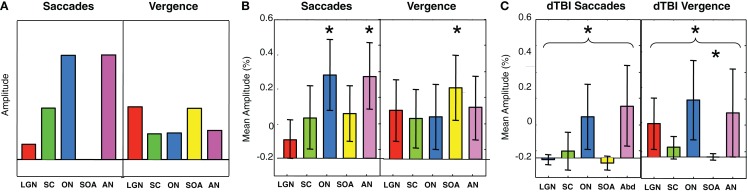
**Predicted (A) and observed (B,C) activation levels in each of five oculomotor nuclei for both saccadic (left-hand panels) and vergence (right-hand panels) eye movements for the control (B) and dTBI (C) groups**. The pairs of brainstem nuclei targeted are as follows: LGN, lateral geniculate nuclei; SC, superior colliculi; ON, oculomotor nuclei; SOAs, supra-oculomotor areas; AN, abducens nuclei. Error bars are 1 SEM. Oculomotor nucleus activation significantly different from 0 at *p* < 0.05 on the *t*-test after correction for multiple planned comparisons are indicated by an asterisk.

To provide hypothesis-based guidance for the planned comparisons in the statistical tests, we need to know what pattern of activations is to be expected for these brainstem nuclei. Predictions for their activation levels can be derived based on the well-established knowledge of their relation to visual, oculomotor, or both visual and oculomotor functions, i.e., these predictions are derived in terms of the combination of visual and/or motor activations in each nucleus in each type of eye movement – saccades and vergence [neurophysiology: (13, 14, 16, 17); fMRI: (9)]. Focusing on the saccades (left-hand panel of Figure 7A), movements of the eyes generate visual signals on the retina. However, saccades have the special property of generating saccadic suppression that is known to block the majority of the visual activation in the LGN, which are purely visual nuclei, and in the visual cortex, making the predicted activation in the LGN low for the saccade condition. Vergence movements, on the other hand, are not subject to saccadic suppression, and hence are predicted to generate higher LGN activation even though the amplitude of the eye movement is substantially smaller. The SC have both visual and oculomotor inputs, so they are only partially subject to saccadic suppression, and should thus follow the same pattern as the LGN for saccades but at higher amplitude. The ON and AN are purely motor controllers for the saccadic movements and are thus not subject to saccadic suppression, so they should show the strongest responses to saccades, but with weaker signals for vergence because the vergence movements are slower (right-hand panel of Figure 7A). (ON activation is not predicted to be zero for vergence because it is the final output nucleus for all medial rectus muscle activations, and should be activated together with the SOA in vergence movements.) The SOA is a purely vergence control nucleus, and are predicted to show responses only for vergence, while the abducens nuclei control the lateral rectus muscles of the two eyes and should be active in both movements, though less so for vergence because it has lower amplitude and velocity.

Based on this logic, the planned comparisons are to test the significance the larger activations relative to 0 (SC, ON, and AN for saccades, LGN and SOA for vergence), for an overall reduction of the combined activation across the five nuclei for the saccade and for the vergence conditions, and individually for the reductions of these five conditions in dTBI relative to control. The statistical significance of these predictions were assessed by the Student’s *t*-test at a criterion of *p* < 0.01, providing Bonferroni correction for multiple applications at *p* < 0.05.

It may be seen in Figure 7B that the activations for the Control group follow approximately the predicted pattern of Figure 7A, with the largest saccadic activations in the ON and AN, and the largest vergence activation in the SOA. Statistically, the two largest saccadic activations and the vergence activation in the SOA are significantly different from 0 (asterisks) though not significantly larger than the other conditions (except for the case of saccadic LGN activation). There is, however, one apparent deviation from the predictions; the saccadic condition shows moderate activation of the vergence center of the SOA (though not statistically significant on the *t*-test at the *p* < 0.01 criterion). This marginal activation may be attributable to the fact that some level of vergence correction was required (as is often reported) when executing the large amplitude saccades (see example in Figure 3).

For the dTBI group (Figure 7C), the activations are clearly reduced overall. Statistical analysis shows that none of the brainstem nuclei activations are significantly different from 0 for the dTBI group, and that the average activation across the five nuclei is significantly reduced relative to controls for the saccadic responses (*p* < 0.01 on the *t*-test). There is also a significant reduction (*p* < 0.01 on the *t*-test) relative to the control group for one case, in particular – the SOA nuclei for vergence movements.

## Discussion

### Oculomotor performance

The oculomotor performance for horizontal *saccades* was in the expected range for the control group and showed a significant deficit in peak velocity and some aspects of the onset latencies and amplitudes for the dTBI group. (Note that the latency and amplitude deficits were each only significant for one eye, but these are different eyes for these two parameters, so we attribute the lateral difference to sampling variations of marginal effects rather than some systematic bias.) Evidently, the saccadic parameters are susceptible to long-term residual effects of the dTBI events and had not fully recovered their original function in terms of oculomotor dynamics. Vergence eye movements also showed a significant slowing of the peak velocities for the dTBI group relative to the control group.

Interestingly, for vergence eye movements, there was a significant slowing of the divergence velocities relative to convergence velocities for both the control and dTBI groups, though no significant difference between the groups in this respect. This slowing of divergence relative to convergence on average replicates the result in studies of normal vergence behavior in Tyler et al. (21, 24).

In summary, the main effect of dTBI on oculomotor performance in the present sample was to produce a significant slowing of both the saccadic and vergence responses relative to the control group.

#### Brainstem Oculomotor Control Mechanisms

We were able to obtain fMRI signals from the three main oculomotor nuclei (ON, AN, and SOA). The control group, showed the expected pattern of strong ON and AN activation for saccades, and strong SOA accompanied by weak ON and AN activation for vergence movements. This is the first study to compare the activation of brainstem ON in dTBI relative to normal, or to measure the activation of the key nuclei of the brainstem oculomotor control network in normals – the ON, the AN, and the SOAs, except for one report of unilateral activation by Alkan et al. (11).

In the dTBI group, the ON and AN showed about a 50% reduction relative to controls, while for the vergence condition, the SOA showed an almost complete reduction relative to controls. These brainstem functional imaging signals are therefore good candidates to serve as non-invasive biomarkers for dTBI deficits in the oculomotor control system.

## Conflict of Interest Statement

The authors declare that the research was conducted in the absence of any commercial or financial relationships that could be construed as a potential conflict of interest.
